# Impact of an Intervention to Control Imipenem-Resistant *Acinetobacter baumannii* and Its Resistance Mechanisms: An 8-Year Survey

**DOI:** 10.3389/fmicb.2020.610109

**Published:** 2021-02-16

**Authors:** Lida Chen, Pinghai Tan, Jianming Zeng, Xuegao Yu, Yimei Cai, Kang Liao, Penghao Guo, Yili Chen, Zongwen Wu, Pinghua Qu, Renxin Cai, Cha Chen, Bin Huang

**Affiliations:** ^1^Department of Laboratory Medicine, The First Affiliated Hospital, Sun Yat-sen University, Guangzhou, China; ^2^Department of Blood Transfusion, China-Japan Friendship Hospital, Beijing, China; ^3^Department of Hematology, Zhujiang Hospital, Southern Medical University, Guangzhou, China; ^4^Department of Laboratory Medicine, The Second Affiliated Hospital, Guangzhou University of Chinese Medicine, Guangzhou, China; ^5^Department of Laboratory Medicine, Guangdong Provincial Hospital of Chinese Medicine, Guangzhou, China

**Keywords:** intervention, *Acinetobacter baumannii*, antibiotic resistance, outbreak, mechanism

## Abstract

**Background:**

This study aimed to examine the impact of an intervention carried out in 2011 to combat multi-drug resistance and outbreaks of imipenem-resistant *Acinetobacter baumannii* (IRAB), and to explore its resistance mechanism.

**Methods:**

A total of 2572 isolates of *A. baumannii*, including 1673 IRAB isolates, were collected between 2007 and 2014. An intervention was implemented to control *A. baumannii* resistance and outbreaks. Antimicrobial susceptibility was tested by calculating minimal inhibitory concentrations (MICs), and outbreaks were typed using pulsed-field gel electrophoresis (PFGE). Resistance mechanisms were explored by polymerase chain reaction (PCR) and whole genome sequencing (WGS).

**Results:**

Following the intervention in 2011, the resistance rates of *A. baumannii* to almost all tested antibiotics decreased, from 85.3 to 72.6% for imipenem, 100 to 80.8% for ceftriaxone, and 45.0 to 6.9% for tigecycline. The intervention resulted in a decrease in the number (seven to five), duration (8–3 months), and departments (five to three) affected by outbreaks; no outbreaks occurred in 2011. After the intervention, only *bla*_AMPC_ (76.47 to 100%) and *bla*_TEM–__1_ (75.74 to 96.92%) increased (*P* < 0.0001); whereas *bla*_GES–__1_ (32.35 to 3.07%), *bla*_PER–__1_ (21.32 to 1.54%), *bla*_OXA–__58_ (60.29 to 1.54%), *carO* (37.50 to 7.69%), and *adeB* (9.56 to 3.08%) decreased (*P* < 0.0001). Interestingly, the frequency of class B β-lactamase genes decreased from 91.18% (*bla*_SPM–__1_) and 61.03% (*bla*_IMP–__1_) to 0%, while that of class D *bla*_OXA–__23_ increased to 96.92% (*P* < 0.0001). WGS showed that the major PFGE types causing outbreaks each year (type 01, 11, 18, 23, 26, and 31) carried the same resistance genes (*bla*_KPC–__1_, *bla*_ADC–__25_, *bla*_OXA–__66_, and *adeABC*), AdeR-S mutations (G186V and A136V), and a partially blocked porin channel CarO. Meanwhile, plasmids harboring *bla*_OXA–__23_ were found after the intervention.

**Conclusion:**

The intervention was highly effective in reducing multi-drug resistance of *A. baumannii* and IRAB outbreaks in the long term. The resistance mechanisms of IRAB may involve genes encoding β-lactamases, efflux pump overexpression, outer membrane porin blockade, and plasmids; in particular, clonal spread of *bla*_OXA–__23_ was the major cause of outbreaks. Similar interventions may also help reduce bacterial resistance rates and outbreaks in other hospitals.

## Background

*Acinetobacter baumannii* is a life-threatening hospital pathogen, causing severe morbidity and mortality, particularly in intensive care units (ICUs) (Jain et al., 2019). Its rate of carbapenem resistance is surprisingly high (Singkham-In and Chatsuwan, 2018), and carbapenem-resistant strains of *A. baumannii*, especially imipenem-resistant *A. baumannii* (IRAB), have caused hospital outbreaks worldwide (Vauchel et al., 2019). To the best of our knowledge, many *A. baumannii* resistance studies have only reported the severity of IRAB resistance, such as mortality and outbreaks (Venditti et al., 2019; Wang et al., 2020). A few measures have been proposed in China (Yu et al., 2019); however, their impact was rarely monitored for a long period.

Hence, the aim of this study was to examine the efficacy of an intervention applied for the prevention and control of IRAB resistance and outbreaks. To discover changes before and after the intervention, we conducted an 8-year continuous survey of IRAB strains with respect to antibiotic resistance, outbreaks, detection of resistance genes, porin channels, and efflux pumps from 2007 to 2014 in a Chinese tertiary hospital.

We believe that similar interventions can help control infections and outbreaks in other hospitals as well, even for other pathogenic bacteria.

## Materials and Methods

### Isolation and Identification of Bacterial Strains

A total of 2572 non-repetitive *A. baumannii* strains, including 1673 IRAB strains, were recovered from samples collected from patients treated in different departments of The First Affiliated Hospital of Sun Yat-sen University, Guangzhou, China between January 2007 and December 2014, and identified using a Vitek2 compact automatic microbiological analysis system with GN colorimetric identification cards (bioMérieux, Marcy l’Etoile, France). These 2572 *A. baumannii* strains were tested for antibiotic resistance; among them, 201 IRAB strains were randomly selected by stratified random sampling method. Of these 201 IRAB strains, the first isolated from different patients each month were randomly selected, and among them a total of 25–26 strains were selected each year (2007–2014). This stratified random sampling method ensured that each IRAB strain had the same probability to be selected, and enabled to monitor statistically the major trends of occurrence of all isolates, reflected by those of the 201 IRAB strains.

These 201 IRAB strains were subjected to pulsed-field gel electrophoresis (PFGE), outbreak analyses, and PCR-based detection of resistance genes. Using whole genome sequencing (WGS), six major PFGE types of outbreak IRAB strains and two control imipenem-sensitive *A. baumannii* (ISAB) strains were sequenced. This study was approved by the Clinical Research and Ethics Committee of The First Affiliated Hospital of Sun Yat-sen University [2019(483)].

### Antimicrobial Susceptibility Testing

Susceptibility testing was performed using AST-GN and AST-GN13 cards on the Vitek2 compact system (bioMérieux, Marcy l’Etoile, France). The microbroth dilution method was used in accordance with the standards published by the Clinical and Laboratory Standards Institute (CLSI). The test agents included imipenem, meropenem, gentamicin, tobramycin, ampicillin/sulbactam, piperacillin/tazobactam, levofloxacin, ciprofloxacin, cefepime, ceftazidime, ceftriaxone, sulfamethoxazole, furantoin, and tigecycline. Based on CLSI clinical breakpoints, isolates were designated as IRAB if imipenem MICs ≥ 8 μg/mL (2018; CLSI Document M100-S28). Quality control for susceptibility testing was performed with *Pseudomonas aeruginosa* ATCC 27853, *Staphylococcus aureus* ATCC 29213, and *Escherichia coli* ATCC 25922, which were purchased from the National Clinical Laboratory Center.

### Pulsed-Field Gel Electrophoresis (PFGE)

The standard protocol of CDC PulseNet^1^ used to subtype *Salmonella* was used for bacterial DNA preparation for PFGE. Agarose plugs containing DNA were digested with 10 U of the restriction enzyme *Apa*I (TaKaRa, Ipswich, MA, United States) for 4 h. Electrophoresis was performed with a 1% SeaKem Gold^®^ agarose gel (Lonza, Basel, Switzerland) in 0.5 × TBE buffer (45 mM Tris, 45 mM boric acid, 1 mM EDTA, pH 8.3) using CHEF Mapper^®^ XA (Bio-Rad Laboratories, Hercules, CA, United States) and alternating pulses at 120°, 6 V/cm, and 5–20 s for 18 h. Genomic DNA of standard *Salmonella* H9812 from the Centers for Disease Control and Prevention was digested with 50 U of *Xba*I and used as a molecular size marker. The interpretation of band patterning was performed according to the criteria of Tenover et al. (1995).

### PCR for Identification of Drug Resistance Genes

Bacterial DNA was extracted from *A. baumannii* isolates by boiling at 100°C for 10 min. PCR was performed for antibiotic resistance-related genes using TaKaRa Ex Taq (Takara Bio Inc., Otsu, Japan) in a 50 μL reaction mixture containing 5 μL 10×buffer, 4 μL of a dNTPs mixture (2.5 mM), 0.25 μL Taq polymerase (5 U/μL), 1 μL forward primer (20 μM), 1 μL reverse primer (20 μM), 1 μL DNA template (20 ng/μL), and nuclease-free water. The PCR cycle consisted of denaturation at 93°C for 2 min, followed by 35cycles of 60 s at 93°C, annealing for 60 s at 55°C, and extension at 72°C for 60 s, with a final extension at 72°C for 10 min. An agarose gel (1%) was used to resolve and detect the PCR products. The primer sequences used for amplification of drug resistance genes are shown in Table 1.

**TABLE 1:** Primer sequences for the amplification of drug resistance genes by PCR.

Genes		Primer sequences		Products (bp)
**β-Lactamases**				
Class A				
	*TEM-1*	F:5′-AGGAAGAGTATGATTCAACA-3′	R:5′-CTCGTCGTTTGGTATGGC-3′	535
	*SHV-1*	F:5′-TGCGCAAGCTGCTGACCAGC-3′	R:5′-TTAGCGTTGCCAGTGCTCG A-3′	305
	*GES-1*	F:5′-ATGCGCTTCATTCACGCAC-3′	R:5′-CTATTTGTCCGTGCTCAGG-3′	864
	*PER-1*	F:5′-AGTCAGCGGCTTAGATA-3′	R:5′-CGTATGAAAAGGACAATC-3′	978
Class B				
	*IMP-1*	F:5′-CGGCCGCAGGAGAGGCTTT-3′	R:5′-AACCAGTTTTGCCTTACCAT-3′	587
	*VIM-1*	F:5′-ATTCCGGTCGGAGAGGTCCG-3′	R:5′-GAGCAAGTCTAGACCGCCCG-3′	633
	*SPM-1*	F:5′-CCTACAATCTAACGGCGACC-3′	R:5′-TCGCCGTGTCCAGGTATAAC-3′	349
	*AIM-1*	F:5′-CTCGGTTTCAGGCCGGAGGA-3′	R:5′-GGGTGACCAGGATGTCGCAGT-3′	478
	*GIM-1*	F:5′-ATTACTTGTAGCGTTGCC-3′	R:5′-CTCTATAAGCCCATTTCC-3′	418
	*NDM-1*	F:5′-GGCGGAATGGCTCATCACGA-3′	R:5′-CGCAACACAGCCTGACTTTC-3′	287
Class C				
	*AMPC*	F:5′-GCCTGGTAAGTATTGGAAAG-3′	R:5′-CCGAAACGGTTAGTTGAGCC-3′	696
	*DHA-1*	F:5′-GCTGCCACTGCTGATAGAA-3′	R:5′-GTTGCCGTCTCCGTAAAG-3′	331
**Class D**				
	*OXA-23*	F:5′-GATGTGTCATAGTATTCGTCG-3′	R:5′-TCACAACAACTAAAAGCACTG-3′	1067
	*OXA-24*	F:5′-GTACTAATCAAAGTTGTGAA-3′	R:5′-TTCCCCTAACATGAATTTGT-3′	800
	*OXA-40*	F:5′-GATGAAGCTCAAACACAGGGTG-3′	R:5′ -TTTCCATTAGCTTGCTCCACC-3′	587
	*OXA-58*	F:5′-AAGTATTGGGGCTTGTGCTG-3′	R:5′-CCCCTCTGCGCTCTACATAC-3′	599
Porin channel	*carO*	F:5′-TATGGATCCTACCAAGCTGAAGT TGGTGGTCG-3′	R:5′-TATGAATTCTTAGAAGCGGTATG CTGCACGAAC-3′	642
Efflux pump	*adeB*	F:5′-GGATTATGGCGACAGAAGGA-3′	R:5′-AATACTGCCGCCAATACCAG-3′	702

### Whole Genome Sequencing (WGS) and Assembly Based Analysis

Genomic DNA was extracted directly from six IRAB isolates of the major outbreak PFGE types and two ISAB isolates as control strains using the MiniBEST kit (Takara Bio Inc.). DNA libraries were prepared using QIAseq FX DNA Library Kits (QIAGEN, Hilden, Germany) and sequenced on an Illumina NextSeq 500 platform (Illumina, San Diego, CA, United States). Paired reads were assembled using SPAdes 3.13.0, and the resulting contigs were annotated in the Prokka website^2^. Assembled contigs were analyzed using the Pasteur Multilocus Sequence Typing (MLST) scheme^3^ and SnapGene 4.3.8.1. SWISS-MODEL^4^ was used for protein modeling.

### Intervention Strategies

**FIGURE 1:**
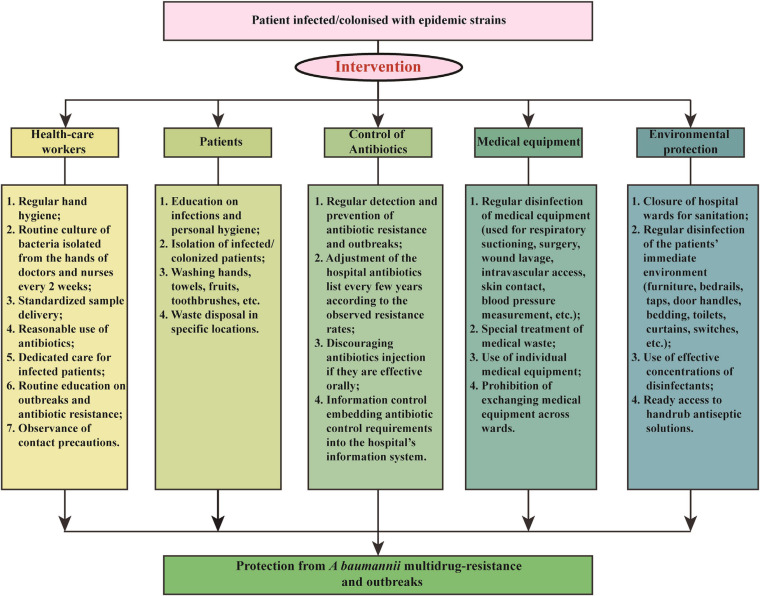
Multicomponent intervention to control *A. baumannii* resistance rates and imipenem-resistant *A. baumannii* outbreaks.

We applied a comprehensive intervention to reduce *A. baumannii* resistance rates and IRAB outbreaks in 2011. The intervention focused of five aspects: health-care workers, patients, antibiotic use, medical equipment, and environmental protection. The details of the measures are shown in Figure 1 and summarized as follows:

(1) Health-care workers: The hospital personnel received lectures on a regular basis about hand hygiene, standardized sample delivery, reasonable use of antibiotics, and dedicated care for infected patients, and routine culture of bacteria isolated from the hands of doctors and nurses every 2 weeks.
(2) Patients: Patients infected or colonized by outbreak bacteria were isolated. In addition, an enhanced personal hygiene, by washing hands, towels, fruits, toothbrushes, etc., and the disposal of waste at specific locations were deemed essential for the containment of *A. baumannii* transmission.
(3) Antibiotic use: Antimicrobials were used more effectively to prevent spread of harmful bacteria and outbreaks, through regular monitoring of antibiotic resistance and adjustment of the hospital antibiotics list every few years according to the observed resistance rates. For example, before the intervention the antibiotics in use were imipenem, meropenem, gentamicin, tobramycin, ampicillin/sulbactam, piperacillin/tazobactam, levofloxacin, ciprofloxacin, cefepime, ceftazidime, ceftriaxone, sulfamethoxazole, and furantoin; however, during the intervention of 2011, meropenem was removed from the hospital antibiotic list due to a rapidly increasing resistance rate, while tigecycline were added to the list. In addition, the injection of antibiotics was avoided if these were effective orally. Finally, an information control system embedding antibiotic control requirements into the hospital’s information system was also applied.
(4) Medical equipment: Special attention was paid to the regular disinfection of medical equipment, such as that used for respiratory suctioning, wound lavage, intravascular access, surgery, skin contact, blood pressure measurement, etc., with 75% ethyl alcohol on a daily basis. Additionally, the use of individual medical equipment and prohibition of exchanging medical equipment across wards also contributed to the control of *A. baumannii* outbreaks.
(5) Environmental protection: Hospital wards were carefully closed for sanitation and effective disinfection of the patients’ immediate environment, including furniture, bedrails, taps, door handles, bedding, toilets, curtains, switches, etc. Moreover, ready access to handrub antiseptic solutions increased the compliance to hand hygiene as well.

### Statistical Analysis

**FIGURE 2:**
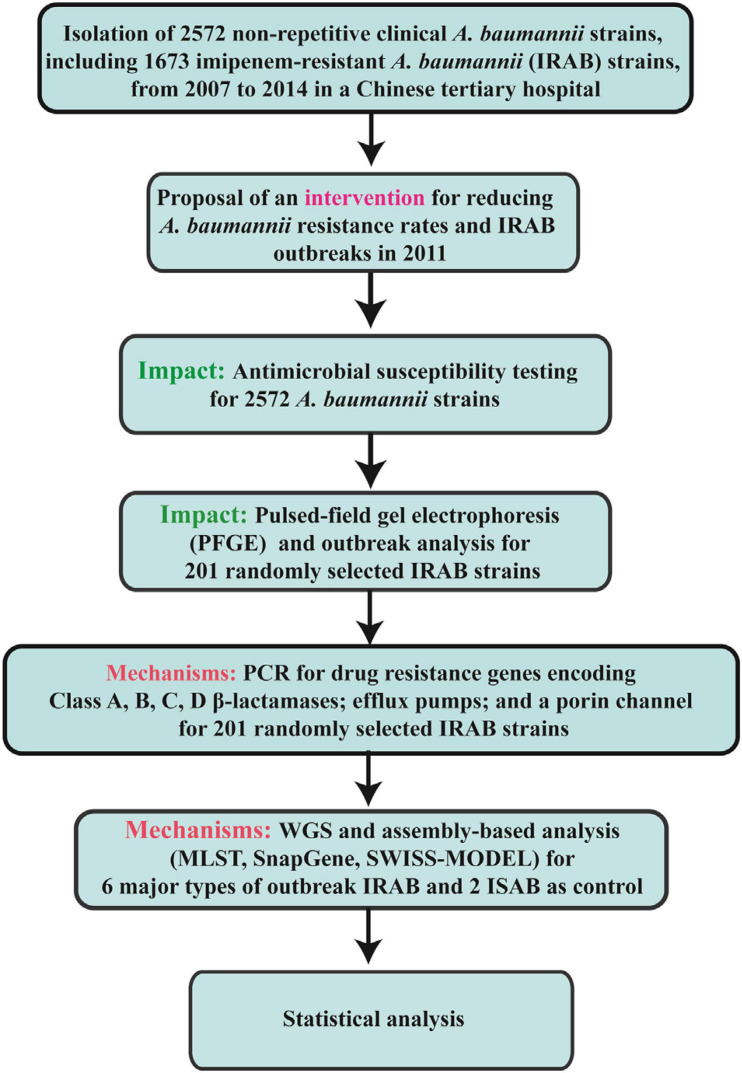
Flow chart of the current research methods.

Continuous variables are presented as means ± standard deviation (SD); categorical variables are shown as numbers and percentages. For continuous variables, comparisons between groups were analyzed by Mann–Whitney test or Student’s *t*-test. For categorical variables, comparisons between groups were analyzed by χ^2^ test. SPSS 19.0 (SPSS Inc., Chicago, IL, United States) was used for data analysis. Results displaying *P* < 0.05 were considered statistically significant. The flow chart of the current research methods are shown in Figure 2.

## Results

### Reduction in Antimicrobial Resistance Rates After Intervention

**FIGURE 3:**
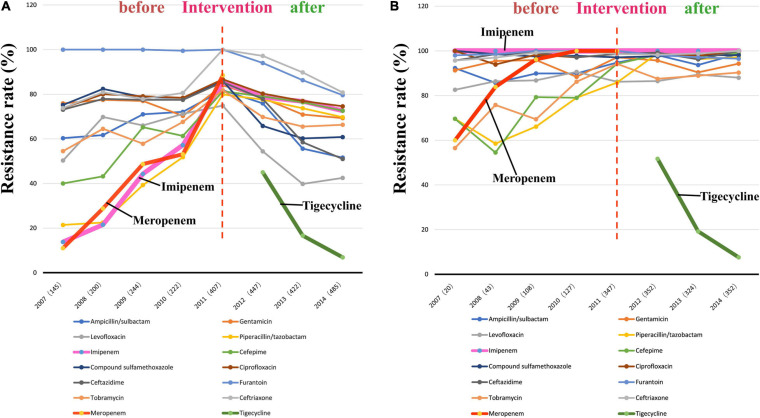
Changes in antibiotic resistance rates during 8 years spanningfrom before to after the intervention. **(A)** Antibiotic resistance rates of 2572 ***A. baumannii*** stains from 2007 to 2014; **(B)** Antibiotic resistance rates of 1673 **IRAB** stains from 2007 to 2014.

The findings of the antibiotic susceptibility testing for all 2572 *A. baumannii* isolates are shown in Table 2. From 2007 to 2010, a total of 811 *A. baumannii* strains were recovered. The rates of resistance to various antibiotics increased every year (Figure 3A and Table 2). Specifically, the resistance rates increased rapidly from 13.8 to 57.2% for imipenem, from 11.0 to 53.1% for meropenem, and from 21.4 to 51.8% for piperacillin/tazobactam. In 2011, *A. baumannii* had the highest resistance rate to imipenem, reaching 85.3%; this trend was also observed for other antibiotics. Interestingly, after reaching its peak in 2011, with the implementation of the intervention in June 2011 (Figure 1), the rates of *A. baumannii* resistance to various antibiotics decreased every year (Figure 3A). Due to the rapidly increasing resistance rate, meropenem was removed from the hospital antibiotics list in 2011, while tigecycline were added to the list after the intervention. From 2011 to 2014, a total of 1761 *A. baumannii* strains were isolated for which the rate of imipenem resistance dropped from 85.3 to 72.6%, that of levofloxacin resistance from 74.9 to 42.5%, that of ceftazidime resistance from 85.4 to 51.0%, that of ceftriaxone resistance from 100.0 to 80.8%, and that of tigecycline resistance from 45.0 to 6.9%. Thus, the resistance rates of *A. baumannii* to almost all the antibiotics in use were reduced after the intervention. The results of antibiotic susceptibility testing for IRAB are shown in Table 2. Unlike *A. baumannii*, the resistance rates of IRAB hardly changed from 2007 to 2014 (Figure 3B and Table 2). A total of 1673 IRAB isolates were resistant to most antibiotics.

**TABLE 2:** Antibiotic resistance rates of 2572 *A. baumannii* stains including 1673 IRAB stains from 2007 to 2014.

Antibiotics agents	2007		2008		2009		2010		2011		2012		2013		2014	
	AB (145) R (%)	IRAB (20) R (%)	AB (200) R (%)	IRAB (43) R (%)	AB (244) R (%)	IRAB (108) R (%)	AB (222) R (%)	IRAB (127) R (%)	AB (407) R (%)	IRAB (347) R (%)	AB (447) R (%)	IRAB (352) R (%)	AB (422) R (%)	IRAB (324) R (%)	AB (485) R (%)	IRAB (352) R (%)
**Carbapenems**																
Imipenem	13.8	100	21.5	100	44.3	100	57.2	100	85.3	100	78.7	100	76.8	100	72.6	100
Meropenem	11.0	60	28.8	83.9	48.7	96.2	53.1	100	88.5	100	**–**	–	**–**	–	**–**	–
**Aminoglycosides**																
Gentamicin	75.9	91.3	77.5	95.5	77.0	95.9	70.3	88.4	84.0	97.1	78.3	95.7	70.9	90.4	69.3	94.3
Tobramycin	54.5	56.5	64.5	75.8	57.8	69.4	67.6	86.2	81.8	94.2	69.8	87.5	65.5	88.9	66.3	90.3
β-lactam antibiotics / enzyme inhibitors																
Ampicillin/sulbactam	60.3	92.3	61.7	85.7	71.0	89.9	72.1	89.9	81.8	94.8	75.9	98.1	55.6	93.7	51.6	98.7
Piperacillin/tazobactam	21.4	69.6	22.5	58.5	39.3	66.1	51.8	79.0	79.9	85.8	77.6	98.9	73.7	96.3	69.7	97.2
**Quinolones**																
Levofloxacin	50.3	82.6	69.8	86.4	66.0	86.8	71.2	90.6	74.9	86.2	54.4	86.5	39.8	89.4	42.5	88.0
Ciprofloxacin	73.8	100	80.0	93.9	79.1	98.3	78.4	97.1	86.7	98.6	80.3	98.9	77.0	97.8	74.6	100
**Cephalosporins**																
Cefepime	40.0	69.6	43.2	54.5	65.2	79.3	61.3	79.0	81.3	94.2	79.0	98.3	76.4	97.5	72.6	99.1
Ceftazidime	73.1	95.7	77.9	98.5	77.5	99.2	77.5	97.1	85.4	98.6	77.9	99.2	58.5	96.2	51.0	100
Ceftriaxone	73.8	95.7	80.9	97.0	77.9	99.2	80.6	100	100	98.6	97.2	98.3	89.8	98.8	80.8	100
**Sulfonamides**																
Sulfamethoxazole	75.2	100	82.4	98.5	78.7	97.5	78.4	97.8	86.0	97.1	65.8	97.6	60.2	98.0	60.8	98.1
**Nitrofurans**																
Furantoin	100	100	100	100	100	100	99.5	100	100	100	94.0	100	86.3	100	79.6	100
**Tetracyclines**																
Tigecycline	**–**	–	**–**	–	**–**	–	**–**	–	**–**	–	45.0	51.7	16.6	19.2	6.9	7.6

*R(%), antibiotic resistance rates (%); AB, *A. baumannii*; IRAB was included in AB in the same year.*

### Pulsed-Field Gel Electrophoresis and Outbreak Analysis From 2007 to 2014

**FIGURE 4:**
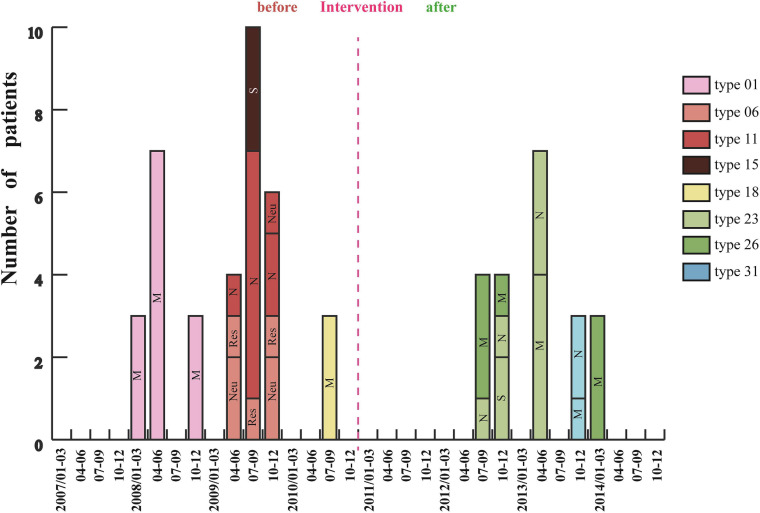
Changes in IRAB outbreaks across 8 years before and after the intervention. Each vertical bar represents an outbreak. M: MICU, Medical Intensive Care Unit; S: SICU, Surgical Intensive Care Unit; N: NICU, Neurology Intensive Care Unit; Neu, Neurology department; and Res, Respiratory department.

Analysis of 201 IRAB strains by PFGE showed that there were seven outbreaks from January 2007 to December 2010 before intervention (Figure 4). Three outbreaks of type 01 occurred between January and December 2008, with a total of 13 cases in the medical ICU (MICU). In 2009 there were three outbreaks: an outbreak of type 06 in the respiratory and neurology departments involving a total of seven cases between April and December; an outbreak of type 11 in the neurology ICU (NICU) and neurology departments with 10 cases from April to December; and an outbreak of type 15 in the surgical ICU (SICU) with three cases from July to September. Furthermore, an outbreak of type 18 involving three cases occurred in MICU from July to September 2010. Almost all outbreak strains (98%) were isolated from sputum specimens. However, after the intervention was applied in 2011 (Figure 1), only five outbreaks were observed from January 2011 to December 2014 (Figure 4). Of these five outbreaks, four cases of type 23 outbreak occurred in the NICU and SICU from July to December 2012, seven cases of type 23 outbreak occurred in the MICU and NICU from April to June 2013, four cases of type 26 outbreak occurred in the MICU from July to December 2012 and three cases from January to March 2014; finally, three cases of type 31 outbreak occurred from October to December 2013 in the MICU and NICU. In summary, after the intervention, the frequency of IRAB outbreaks, the duration of each outbreak, and the number of outbreak strains decreased notably; in addition, there was no outbreak in 2011, and the major PFGE types of outbreak strains before and after the intervention were also different.

### Distribution of Genes Encoding β-Lactamases, Porin Channels, and Efflux Pumps

The distribution of genes encoding β-lactamases, porin channels, and efflux pumps in 201 randomly selected IRAB strains is shown in Table 3. Screening of resistance genes showed that before the intervention, from 2007 to 2010, the most prevalent β-lactamase-coding resistance gene was *bla*_*AMPC*_ (76.47%) of class C, followed by *bla*_*TEM–*__1_ (75.74%) of class A. Moreover, the IRAB isolates were found to harbor *bla*_*IMP–*__1_ (61.03%), *bla*_*VIM–*__1_ (40.44%), *bla*_*SPM–*__1_ (91.18%), *bla*_*AIM–*__1_ (43.38%), *bla*_*GIM–*__1_ (14.71%), and *bla*_*NDM–*__1_ (1.47%). However, the prevalence of the resistance genes from the 2011–2014 periods changed after the intervention. Among the IRAB strains, the prevalence of *bla*_*TEM–*__1_ (96.92%), *bla*_*A*__*MPC*_ (100.00%), and *bla*_*OXA–*__23_ (96.92%) had significantly increased (*P* < 0.001). Notably, the prevalence of the *bla*_*OXA–*__23_ gene had significantly increased from 23.53% to 96.92% (*P* < 0.001), whereas that of *bla*_*GES–*__1_ (3.07%), *bla*_*PER–*__1_ (1.54%) of class A, *bla*_*DHA*_ (1.54%) of class C, *bla*_*OXA–*__58_ (1.54%) of class D, and *carO* (7.69%) had significantly decreased (*P* < 0.0001). In addition, the prevalence of class B genes, such as *bla*_*SPM–*__1_, *bla*_*IMP–*__1_, *bla*_*VIM–*__1_, *bla*_*AIM–*__1_, and *bla*_*GIM–*__1_, decreased to negligible levels (*P* < 0.0001).

**TABLE 3:** Distribution of resistance genes in 201 IRAB isolates from 2007 to 2014.

Genes	2007–2010 IRAB (*n* = 136) Prevalence rate (%) before the intervention	2011-2014 IRAB (*n* = 65) Prevalence rate (%) after the intervention	χ^2^	*P-*value
**β-lactamases Class A**				
*bla*_*TEM–1*_	75.74 (103/136)	96.92 (63/65)	13.729	<0.0001
*bla*_*SHV–1*_	1.47 (2/136)	0 (0/65)	0.965	1.000
*bla*_*GES–1*_	32.35 (44/136)	3.07 (2/65)	21.359	<0.0001
*bla*_*PER–1*_	21.32 (29/136)	1.54 (1/65)	13.558	<0.0001
**Class B**				
*bla*_*IMP–1*_	61.03 (83/136)	0 (0/65)	67.572	<0.0001
*bla*_*VIM–1*_	40.44 (55/136)	0 (0/65)	36.189	<0.0001
*bla*_*SPM–1*_	91.18 (124/136)	0 (0/65)	154.704	<0.0001
*bla*_*AIM–1*_	43.38 (59/136)	0 (0/65)	39.915	<0.0001
*bla*_*GIM–1*_	14.71 (20/136)	0 (0/65)	10.615	<0.0001
*bla*_*NDM–1*_	1.47 (2/136)	0 (0/65)	0.965	1.000
**Class C**				
*bla*_*AMPC*_	76.47 (104/136)	100 (65/65)	0.965	<0.0001
*bla*_*DHA–1*_	19.85 (27/136)	1.54 (1/65)	12.304	<0.0001
**Class D**				
*bla*_*OXA–23*_	23.53 (32/136)	96.92 (63/65)	95.046	<0.0001
*bla*_*OXA–24*_	2.21 (3/136)	0 (0/65)	1.456	0.552
*bla*_*OXA–40*_	0.74 (1/136)	0 (0/65)	0.480	1.000
*bla*_*OXA–58*_	60.29 (82/136)	1.54 (1/65)	62.631	<0.0001
**Porin channel**				
*carO*	37.5 (51/136)	7.69 (5/65)	19.442	<0.0001
**Effux pump**				
*adeB*	9.56 (13/136)	3.08 (2/65)	1.819	0.151

### Whole Genome Sequencing (WGS) and Assembly Based Analysis

Whole genome sequencing was used to detect the major outbreak PFGE types (Figure 4) of IRAB strains before the intervention (SQ001-type 01, SQ002-type 11, and SQ003-type 18), after the intervention (SQ081-type 31, SQ092-type 23, and SQ093-type 26), and the control strains SQ082-ISAB (S) and SQ080-ISAB (MDR) to analyze the resistance mechanisms of IRAB. The control strain SQ082-ISAB (S) was sensitive to all antibiotics. SQ080-ISAB (MDR-AB) and SQ081-IRAB belonged to the same PFGE type 31; SQ080 was resistant to multi-antibiotics, but sensitive to imipenem, while SQ081 was resistant to both multi-antibiotics and imipenem.

**FIGURE 5:**
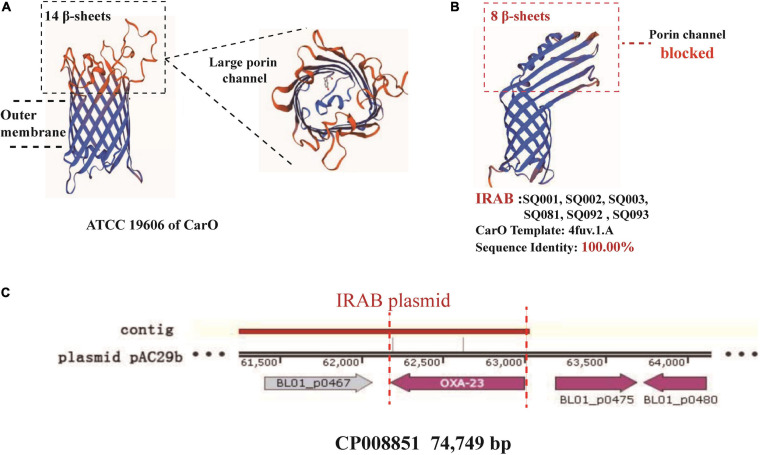
SWISS-model for porin permeability to imipenem and plasmid harboring *bla*_*OXA–23*_ in IRAB isolates. **(A)** 3D model for CarO of ATCC 19606 with 14 β-sheets, which form a large porin channel for imipenem to pass through; **(B)** 3D model for CarO of IRAB with 8 β-sheets, which block the porin channel to hinder the passage of imipenem; the sequence identity with the template 4fuv.1.A was 100%; **(C)** plasmid harboring *bla*_*OXA–23*_ in IRAB isolates.

The study of the virulence mechanism of outbreak strains by WGS revealed that these differed before and after the intervention (Table 4). For instance, the *bla*_*OXA–*__23_ gene, coding for a class D β-lactamase, was present in IRAB (SQ081-type 31, SQ092-type 23, and SQ093-type 26) isolated after the intervention, but not in those IRAB isolates (SQ001-type 01, SQ002-type 11, and SQ003-type 18) that had been collected before the intervention. Second, the six IRAB isolates collected before (SQ001-type 01, SQ002-type 11, and SQ003-type 18) and after (SQ081-type 31, SQ092-type 23, and SQ093-type 26) the intervention showed some similarities in multiple drug resistance mechanisms, differing from ISAB (S) (SQ082): ① The six IRAB isolates were all ST2 by MLST analysis, while ISAB (S) (SQ082) was ST218; ② Compared to ISAB (S) (SQ082), these IRAB carried a wide variety of antibiotic enzymes: their β-lactamase genes included *bla*_*KPC–*__1_ in class A, *bla*_*ADC–*__25_ in class C, and *bla*_*OXA–*__66_ in class D; ③ Unlike ISAB(S) (SQ082), these IRAB displayed genes encoding aminoglycoside-modifying enzymes including *ant(3′)*, *ant(3′′)*, and *aph;* ④ Among efflux pumps, although the *adeABC* operon could be detected in IRAB, *adeC* could not be detected in ISAB(S) (SQ082). AdeR carried a A136V mutation, and AdeS a G186V mutation in IRAB, but AdeR-S showed no mutations in ISAB(S). IRAB also presented the genes *msrE* and *tetA*, encoding efflux pumps, while ISAB(S) (SQ082) did not; ⑤ With respect to the outer membrane porin permeability of antibiotics, the amino acid sequence of the CarO of IRAB (SQ001-type 01, SQ002-type 11, SQ003-type 18, SQ081-type 31, SQ092-type 23, and SQ093-type 26) matched that of the sequence under NCBI accession number WP_004714722.1, while that of ISAB(S) (SQ082) matched the sequence of the NCBI accession number WP_ 000866519.1. The SWISS-MODEL for CarO shows that the upper part of ATCC 19606 (Figure 5A) consists of a large channel formed by 14 β-sheets, while that of IRAB is formed by 8 β-sheets, leading to partial blockade of the channel (Figure 5B). The similarity between the CarO of IRAB and the CarO template 4fuv.1.A was 100% (Figure 5B), whereas the similarity between the CarO of ISAB (S) (SQ082) and the template 4fuv.1.A was only 75.93%; ⑥ Plasmid analysis revealed that the plasmid carried by IRAB (SQ081-type 31, SQ092-type 23, and SQ093-type 26) was similar to the plasmid pAC29b (NCBI registration number CP008851). The comparison of plasmids and contigs using the SnapGene software revealed that the IRAB plasmid covered the full length of *bla*_*OXA–*__23_ on plasmid pAC29b, which contained the *bla*_*OXA–*__23_ gene, indicating that *bla*_*OXA–*__23_ was present on the plasmid of IRAB after the intervention (Figure 5C).

**TABLE 4:** Whole genome sequencing for major outbreak types before and after the intervention.

Strains	Control		Before			After		
	SQ082	SQ080	SQ001	SQ002	SQ003	SQ081	SQ092	SQ093
Resistance	ISAB(S)	ISAB(MDR)	IRAB	IRAB	IRAB	IRAB	IRAB	IRAB
Accession No.	JADQAD 000000000	JADPZY 000000000	JADQAA 000000000	JADPZZ 000000000	JADQAG 000000000	JADQAC 000000000	JADQAE 000000000	JADQAF 000000000
PFGE	Type 35	Type 31	Type 01	Type 11	Type 18	Type 31	Type 23	Type 26
MLST	218	2	2	2	2	2	2	2
**β-Lactamases**								
Class A	*bla*_*TEM–4*_	*bla*_*TEM–4*_	*bla*_*TEM–4*_	*bla*_*TEM–4*_	*bla*_*TEM–4*_	*bla*_*TEM–4*_	*bla*_*TEM–4*_	*bla*_*TEM–4*_
	*bla*_*TEM–104*_	*bla*_*TEM–104*_	*–*	*–*	*–*	*–*	*–*	*–*
	*bla*_*TEM–143*_	*bla*_*TEM–143*_	*–*	*–*	*–*	*–*	*–*	*–*
	*–*	*–*	***bla*_*KPC–*_***-*	***bla*_*KPC–1*_***-*	***bla*_*KPC–1*_***-*	***bla*_*KPC–1*_***-*	*–*	*–*
Class B	*–*	*–*	*–*	*–*	*–*	*–*	*–*	*–*
Class C	*bla*_*ADC–80*_	***bla*_*ADC–25*_**	***bla*_*ADC–25*_**	***bla*_*ADC–25*_**	***bla*_*ADC–25*_**	***bla*_*ADC–25*_**	***bla*_*ADC–25*_**	***bla*_*ADC–25*_**
Class D	*–*	*–*	*–*	*–*	*–*	***bla*_*OXA–23*_**	***bla*_*OXA–23*_**	***bla*_*OXA–23*_**
	*–*	***bla*_*OXA–66*_**	***bla*_*OXA–66*_**	***bla*_*OXA–66*_**	***bla*_*OXA–66*_**	***bla*_*OXA–66*_**	***bla*_*OXA–66*_**	***bla*_*OXA–66*_**
Aminoglycoside	*AAC(3)*	*AAC(3)*	*AAC(3)*	*AAC(3)*	*AAC(3)*	*AAC(3)*	*–*	*–*
modifying	*AAC(6′)-Ib8*	*AAC(6′)-Ib8*	*AAC(6′)-Ib8*	*–*	*–*	*AAC(6′)-Ib8*	*–*	*–*
enzymes	*AddA*	*AddA*	*AddA*	*–*	*–*	*AddA*	*–*	
	*ANT*	*ANT*	*ANT*	*ANT*	*ANT*	*ANT*	*–*	*–*
	*–*	***aph***	***aph***	***aph***	***aph***	***aph***	***aph***	
	*–*	*–*	*–*	***ant(3′)***	***ant(3′)***	*–*	***ant(3′)***	***ant(3′)***
**Efflux pumps**								
adeABC	*adeAB*	*adeABC*	*adeABC*	*adeABC*	*adeABC*	*adeABC*	*adeABC*	*adeABC*
adeFGH	+	+	+	+	+	+	+	+
adeIJK	+	+	+	+	+	+	+	+
AdeR mutation	–	A136V	A136V	A136V	A136V	A136V	A136V	A136V
AdeS mutation	–	G186V	G186V	G186V	G186V	G186V	G186V	G186V
others	*abeM*	*abeM*	*abeM*	*abeM*	*abeM*	*abeM*	*abeM*	*abeM*
	*abeS*	*abeS*	*abeS*	*abeS*	*abeS*	*abeS*	*abeS*	*abeS*
	*emrA*	*–*	*–*	*–*	*–*	*emrA*	*–*	*–*
	*emrB*	*–*	*–*	*–*	*–*	*–*	*–*	*–*
	*–*	*msrE*	*msrE*	*–*	*msrE*	*–*	*msrE*	*msrE*
	*–*	*tet(A)*	*tet(A)*	*–*	*tet(A)*	*tet(A)*	*tet(A)*	*tet(A)*
**porin**								
CarO (NCBI)	+	+	+	+	+	+	+	+
	WP_	WP_	WP_	WP_	WP_	WP_	WP_	WP_
	000866519.1	004714722.1	004714722.1	004714722.1	004714722.1	004714722.1	004714722.1	004714722.1

*The accession numbers of JAD…originate from database https://www.ncbi.nlm.nih.gov/assembly/, and the accession numbers of WP_…originate from database https://www.ncbi.nlm.nih.gov/protein/.*

### Potential Resistance Mechanism of IRAB Before and After the Intervention

**FIGURE 6:**
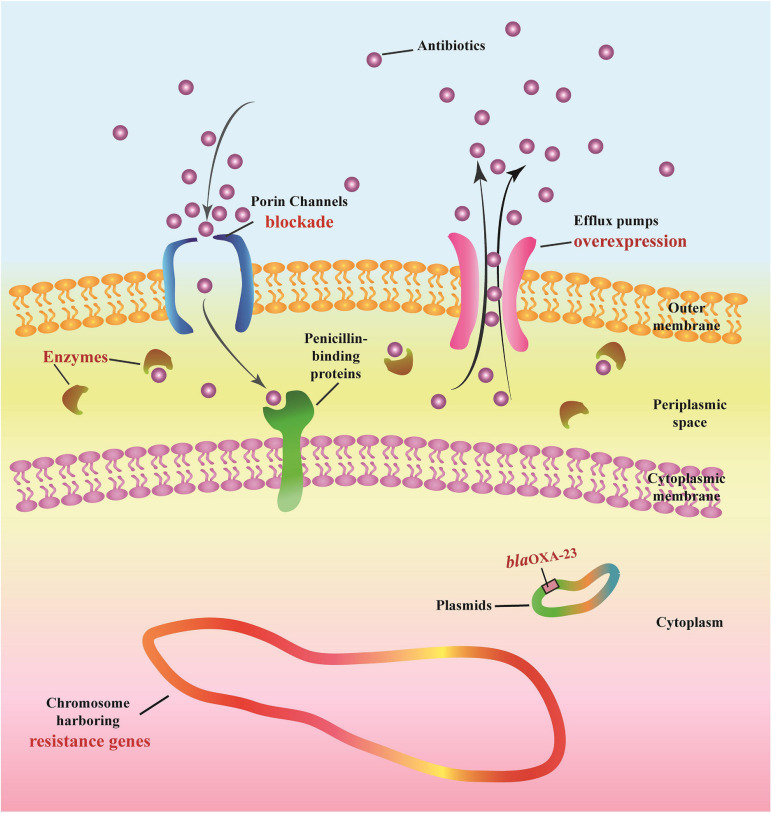
Potential mechanisms of antimicrobial resistance in IRAB.

The following multi-drug resistance mechanisms were observed in IRAB isolates before and after the intervention. First, common mechanisms included (1) the partial blockade of the CarO porin channel, which reduced the antibiotic permeability of IRAB (Table 3 and Figure 5B); (2) the possible overexpression of the efflux pump AdeABC, owing to AdeR-S mutations (Tables 3, 4), which enhanced the antibiotic efflux capacity of IRAB; (3) the presence of enzymes residing in the periplasmic space, such as β-lactamases (encoded by *bla*_*TEM–*__1_, *bla*_*TEM–*__4_, *bla*_*GES–*__1_, and *bla*_*PER–*__1_ in class A; *bla*_*AMPC*_, *bla*_*DHA*_, and *bla*_*ADC–*__25_ in class C; and *bla*_*OXA–*__66_ in class D) (Tables 3, 4) and aminoglycoside-modifying enzymes [encoded by *ant(3′)*, *ant(3′′)*, *aph*] (Table 4) which can degrade various antibiotics in IRAB. Second, WGS highlighted the occurrence of different mechanisms before and after intervention: in particular, among the enzymes residing in the periplasmic space, the types of β-lactamases changed, as *bla*_*OXA–*__23_ in class D was only detected on the bacterial plasmid after the intervention (Table 4). Therefore, the plasmids harboring *bla*_*OXA–*__23_ may be the major cause of IRAB outbreak after the intervention (Table 4 and Figures 4, 5C). The multi-drug resistance mechanisms of IRAB after the intervention are shown in Figure 6.

Imipenem-resistant *Acinetobacter baumannii* contains an outer membrane, periplasmic space, cytoplasmic membrane, cytoplasm, chromosome, and plasmids. Antibiotics must cross the outer membrane through porin channels (outer membrane proteins such as CarO) to reach the periplasmic space and then bind to the final targets of penicillin-binding proteins located at the level of the cytoplasmic membrane. Antibiotics can be degraded by enzymes (e.g., β-Lactamases of Class A, B, C, and D), and can also be actively expelled from the bacterial structure through efflux pumps (e.g., AdeABC and AdeR-S).

### Impact of the Intervention

The intervention triggered many positive effects, that we describe as follows: ① the resistance rates of *A. baumannii* decreased after the intervention; ② the intervention resulted in a decrease in the number and duration of outbreaks, as well as in the number of departments affected by of outbreaks; ③ the frequency of most resistance genes decreased after the intervention; ④ the resistance mechanisms characterizing IRAB both before and after the intervention included porin channel blockade, efflux pump overexpression, and chromosomes harboring resistance genes and various enzymes; conversely, the frequent genes encoding β-lactamases passed from genes encoding class B β-lactamases to *bla*_*OXA–*__23_ after the intervention.

## Discussion

### This Study Proposed a Systemic Intervention to Control *A. baumannii* Resistance and IRAB Outbreaks

Imipenem-resistant *Acinetobacter baumannii* is the most important group of bacteria associated with hospital-acquired infections and outbreaks worldwide (Xie et al., 2018). Several control measures have been applied against IRAB (Birgand et al., 2015, 2016), and some articles display various degrees of severity and prevalence of IRAB over many years (Du et al., 2019; Park et al., 2019). However, few studies similar to the present study have been published. In the present study, we proposed a systemic control intervention and also investigated the changes in severity and prevalence of resistance mechanisms of IRAB for a long time (8 years). The aim of this study was to characterize the impact of an intervention to control resistance and outbreaks of IRAB and to explore its resistance mechanisms through an 8-year survey. We hope that our results can help hospitals in other countries to prevent and control *A. baumannii* resistance and IRAB outbreaks.

### The Intervention Reduced the Antibiotic Resistance Rate of *A. baumannii* and the Occurrence Rate of IRAB

Before the intervention, the drug resistance rate of *A. baumannii* increased every year, and the resistance rate for imipenem increased from 13.8% to 85.3%. During the intervention in 2011, meropenem was removed from the hospital antibiotics list due to a rapid growth of resistance rate, while tigecycline were added. After the intervention, the resistance rates of *A. baumannii* to various antibiotics decreased significantly every year. However, IRAB isolates were almost completely resistant to other antibiotics, and the resistance rates of IRAB to other antibiotics did not change significantly during the 8 years of the survey, which suggests that the intervention could effectively prevent and control the resistance rates of *A. baumannii* to various antibiotics and to imipenem, but had limited effect on *A. baumannii* that is already resistant to imipenem (IRAB). This could be due to the complex resistance mechanisms characterizing IRAB reported by Lee et al. (2017); indeed, once *A. baumannii* becomes IRAB, it might be difficult to reduce the drug resistance rates due to the emergence of complex resistance mechanisms to many antibiotics.

### The Intervention Reduced the Frequency, Duration, and Isolate Number of IRAB Outbreaks

Pulsed-field gel electrophoresis analysis showed that the frequency of IRAB outbreaks before the intervention was 2–3 times per year, which decreased to almost once a year after the intervention. The duration of the IRAB outbreak was shortened from 8 to 3 months. The outbreaks occurred in the MICU, SICU, NICU, and respiratory and neurology departments before the intervention, but only in the MICU, SICU, and NICU after the intervention. Daniel et al. (2019) reported that MICU, SICU, and NICU were the key departments of IRAB outbreaks, consistent with our findings. The number of IRAB strains per outbreak was reduced from 11 to 3 isolates. Notably, there was no outbreak in 2011, the first year after implementing the intervention, which could be attributed to the replacements in the hospital antibiotic list and other control measures in the intervention. However, new IRAB strains that were resistant to new antibiotics and selection pressures caused new outbreaks after 2011. Therefore, regular replacement of antibiotics to prevent the emergence of new IRAB strains is also a key feature of this intervention.

### PCR-Based Mechanistic Studies Showed That Changing Antibiotics in the Intervention May Lead to Changes in IRAB Resistance Mechanisms

Imipenem-resistant *Acinetobacter baumannii* resistance was the result of a combination of multiple resistance mechanisms. PCR analysis showed that genes encoding β-lactamases, such as *bla*_*AMPC*_ and *bla*_*TEM–*__1_were the major resistance genes of IRAB before and after the intervention. Before the intervention, *bla*_*SPM–*__1_ and *bla*_*IMP–*__1_ in class B were detected at a frequency of 91.18 and 61.03%, respectively, while *bla*_*OXA–*__23_ in class D accounted for only 23.53%. However, after the intervention, the frequency of genes in class B decreased to 0.00%, but that of *bla*_*OXA–*__23_ of class D increased to 96.92%, suggesting that there was clonal spread (or horizontal spread) of *bla*_*OXA–*__23_ after 2011. Harboring the *bla*_*OXA–*__23_ gene may have been one of the main resistance mechanisms of IRAB from 2011 to 2014. In the intervention, meropenem was replaced with tigecycline. Kulengowski et al. (2019) reported that *bla*_*VIM–*__1_ of class B was related to meropenem resistance, while Savari et al. (2017) reported that *bla*_*OXA–*__23_ and overexpression of the AdeABC efflux pump were associated with tigecycline resistance. Therefore, the significant differences in frequency between class B genes and *bla*_*OXA–*__23_ before and after the intervention may be due to the replacement of antibiotics, which changed the selection pressure of *A. baumannii*, leading to changes in the drug resistance mechanism. The gene *adeB* encoding an efflux pump and the gene *carO* encoding a porin channel were also analyzed before and after the intervention. Therefore, multiple drug resistance mechanisms mediated widespread IRAB resistance.

### WGS-Based Mechanistic Analysis Confirmed That IRAB Has a Combination of Resistance Mechanisms, and That Plasmids Harboring *bla*_*OXA–*__23_ May Lead to New Outbreaks After the Intervention

Whole genome sequencing showed that IRAB had multiple drug resistance mechanisms compared to ISAB (S) (SQ080): (1) There are many types of enzymes for modification of antibiotics, such as β-lactamases and aminoglycoside-modifying enzymes. First, the production of β-lactamases is a major antibiotic resistance mechanism in *A. baumannii*; these enzymes inactivate β-lactam, and comprise four molecular classes: A, B, C, and D (Sawa et al., 2020). In this study, unlike ISAB (S)(SQ082), IRAB contained *bla*_*KPC –*__1_ of class A, *bla*_*ADC–*__25_ of class C, and *bla*_*OXA–*__23_, and *bla*_*OXA–*__66_ of class D. Class A β-lactamases degrade early generation cephalosporins (Sawa et al., 2020); class C β-lactamases can confer resistance to cephamycins (cefoxitin and cefotetan), penicillins, and cephalosporins (Uddin et al., 2018); class D β-lactamases, also called OXAs (oxacillinases), hydrolyze oxacillin, and the presence of carbapenem-hydrolyzing class D β-lactamases is a major carbapenem resistance mechanism in *A. baumannii* (Stewart et al., 2019). Unlike the serine-dependent β-lactamases (classes A, C, and D), class B β-lactamases are metallo-β-lactamases that require zinc or another heavy metal for catalysis (Amin et al., 2019). However, genes of class B were not detected in IRAB or ISAB, indicating that class B β-lactamases did not cause IRAB resistance. Second, aminoglycoside-modifying enzymes are classified into acetyltransferases, adenyltransferases, and phosphotransferases (Sheikhalizadeh et al., 2017). In the present study, IRAB had *ant(3′)*, *ant(3′′)*, and *aph* genes, which conferred resistance to aminoglycosides. (2) Efflux pumps are associated with resistance against many different classes of antibiotics, such as imipenem and tigecycline, in *A. baumannii* (Knight et al., 2018). AdeABC is associated with aminoglycoside resistance and with decreasing susceptibility to tigecycline and fluoroquinolone antibiotics (Yoon et al., 2013). The *adeABC* gene was detected in IRAB, but *adeC* was not detected in ISAB, suggesting that efflux pumps of IRAB are more effective than those of ISAB. Notably, mutations in the AdeR-S two-component system lead to the overexpression of AdeABC (Yang et al., 2019). In IRAB, AdeR had a G186V mutation, and AdeS had an A136V mutation, but AdeR-S of ISAB had no mutation, suggesting overexpression of efflux pumps that are more effective than those of ISAB for antibiotic efflux. (3) To investigate the porin channel mechanism, the 3D structure of CarO was modeled using the SWISS-MODEL software. Compared to the structure of CarO from the standard strain ATCC 19606, it was found that the upper part of CarO from ATCC 19606 was a large channel formed by 14 β-sheets, while the upper part of CarO from IRAB in this study was only a small channel formed by 8 β-sheets; such change in the tertiary structure of the IRAB CarO suggested that the porin channel was partly blocked. In addition, the structure of CarO from IRAB elaborated in this study was 100% identical to that of the template 4fuv.1.A in SWISS-MODEL, representing a CarO channel with low permeability to imipenem. Zhu et al. (2019) reported that a partial blockade of the CarO porin channel can reduce the penetration of antibiotics into bacteria, leading to drug resistance, consistent with the results of this study. (4) Plasmid mechanistic analysis revealed that *bla*_*OXA–*__23_ was present on the plasmid of IRAB after the intervention, and WGS showed that *bla*_*OXA–*__23_ transmitted drug resistance through the plasmid and became the major reason for IRAB prevalence and outbreak after the intervention. Xiong et al. (2015) reported that changes in drug resistance mechanisms of bacteria may be due to different environmental selection pressure; therefore, with our intervention we might have induced a modification of the prevalent drug resistance mechanisms through a variety of control measures and adjustment of antibiotics use. For example, during the intervention meropenem, showing a fast-growing resistance rate, was discontinued while tigecycline was adopted. Such changes in environmental selection pressure may have reduced the prevalence of class B β-lactamases; consequently, *bla*_*OXA–*__23_ began to spread and became the main epidemic factor after the intervention. This phenomenon could also explain why the outbreak did not occur throughout 2011 after applying the intervention, but started in July 2012: indeed, *A. baumannii* may have adapted, and major strains harboring the plasmid bearing *bla*_*OXA–*__23_ may have been selected under the new environmental pressure throughout 2011. Therefore, the resistance to new antibiotics must be regularly monitored, and these should be replaced to prevent the emergence of new resistant strains in each new round of intervention. The present study demonstrated that strengthening the management of health-care workers, patients, medical equipment, and environmental protection is an effective measure against IRAB outbreaks. In addition, modifying the antibiotics list of the hospital according to the results of drug sensitivity is also an effective measure against IRAB resistance, for example by removing ampicillin, furantoin, and ceftriaxone to exhibit high resistance rates, and adding ceftazidime/avibactam (CZA). In fact, Norelle et al. (2018) reported that CZA exerted good inhibitory effects on bacteria carrying *bla*_*OXA–*__23_.

## Conclusion

In this study we proposed and applied a systemic intervention to control the antibiotic resistance of *A. baumannii* and the outbreak of IRAB, which included five aspects: health-care workers, patients, antibiotic use, medical equipment, and environmental protection. An 8-year survey has proven the effectiveness of the intervention, which led to a significant reduction in resistance rates; a decrease in the number, duration, and number of departments affected by outbreaks; and a reduced frequency of most resistance genes. The resistance mechanisms of IRAB include porin channel blockade, efflux pump overexpression, various enzymes, chromosomes harboring resistance genes, and *bla*_*OXA–*__23_ on plasmids, which was the major reason for outbreaks after the intervention. As part of our intervention strategy, new rounds of interventions should be taken every few years to prevent the emergence of new resistant strains. We hope that this intervention strategy can help other countries and regions control the resistance of *A. baumannii* and outbreaks of IRAB.

## Data Availability Statement

The original contributions presented in the study are included in the article/supplementary material, further inquiries can be directed to the corresponding author/s.

## Ethics Statement

This study was approved by the Clinical Research and Ethics Committee of The First Affiliated Hospital of Sun Yat-sen University [2019(483)].

## Author Contributions

LC, PT, JZ, XY, YC, KL, PG, YC, ZW, PQ, and RC conducted the experiments. LC wrote the manuscript. CC and BH edited the manuscript. All the authors contributed to the article and approved the submitted version.

## Conflict of Interest

The authors declare that the research was conducted in the absence of any commercial or financial relationships that could be construed as a potential conflict of interest.

## Footnotes

1 http://www.cdc.gov/pulsenet/

2 http://www.vicbioinformatics.com/software.prokka.shtml

3 www.genomicepidemiology.org

4 https://swissmodel.expasy.org
